# Mindestlohnbetriebe in der zweiten Corona-Welle

**DOI:** 10.1007/s10273-021-3028-9

**Published:** 2021-10-23

**Authors:** Christian Kagerl, Clemens Ohlert

**Affiliations:** 1 Forschungsgruppe des Direktors, Institut für Arbeitsmarkt- und Berufsforschung (IAB), Regensburger Straße 104, 90478 Nürnberg, Deutschland; 2 c/o Bundesanstalt für Arbeitsschutz und Arbeitsmedizin, Geschäfts- und Informationsstelle für den Mindestlohn, Nöldnerstraße 40-42, 10317 Berlin, Deutschland

**Keywords:** J23, J38, M54

## Abstract

Die Mindestlohnbetriebe können es in der Corona-Pandemie aus verschiedenen Gründen besonders schwer haben. Wir stellen fest, dass Mindestlohnbetriebe zu Beginn 2021 häufiger von der Corona-Pandemie betroffen waren als die übrige Wirtschaft, was auf die Branchenzusammensetzung der Mindestlohnbetriebe und nicht auf den Mindestlohn selbst zurückzuführen ist. Die Ergebnisse zeigen auch, dass Mindestlohnbetriebe häufiger Kurzarbeit einsetzen als andere Betriebe. Auch das Arbeitsvolumen wird in Mindestlohnbetrieben häufiger als in anderen Betrieben durch den Abbau von Überstunden reduziert. Zudem werden Lohnerhöhungen und Sonderzahlungen häufiger gekürzt oder ausgesetzt.

## Abstract (en)

For a number of reasons, businesses affected by minimum wage may have a particularly hard time during the coronavirus pandemic. We have found that minimum wage establishments were more frequently affected by the Corona pandemic than the rest of the economy at the beginning of 2021 due to the industry composition of these establishments rather than to minimum wage itself. The findings also indicate that minimum-wage establishments use short-time work more often and that the volume of work is reduced more frequently than in other establishments by cutting back on overtime. Finally, salary increases and special payments are also reduced or suspended more frequently.

Die Einführung des gesetzlichen Mindestlohns fand in einer wirtschaftlich sehr guten Lage statt. Dies hat sich mit Beginn der Corona-Krise, die in Deutschland seit dem zweiten Quartal 2020 einsetzte, geändert. Es kam zu Unterbrechungen globaler Lieferketten, Personalausfällen und Einschränkungen des Betriebs bzw. Betriebsverboten in Lockdowns. Entgegen anfänglichen Hoffnungen waren die wirtschaftlichen Auswirkungen der Corona-Pandemie nicht nur von kurzer Dauer. Zu Beginn 2021 befand sich Deutschland auf dem bisherigen Höhepunkt der Pandemie, die zu starken Einschränkungen des öffentlichen Lebens und der betrieblichen Aktivitäten von Anfang November 2020 bis ins Frühjahr 2021 führte.

Die Erhöhungen des gesetzlichen Mindestlohns zur Zeit der Corona-Pandemie waren moderat.[1] Anfang 2021 betrug der gesetzliche Mindestlohn 9,50 Euro. Die nächste deutliche Erhöhung des Mindestlohns, dann auf 10,45 Euro, steht erst zum Juli 2022 an. Dennoch gibt es eine Reihe von Gründen für die Annahme, dass es vom Mindestlohn betroffene Betriebe in der Corona-Pandemie besonders schwer haben könnten. Dies könnte z. B. daran liegen, dass diese Betriebe häufig in Wirtschaftsbereichen aktiv sind, die von der Pandemie bzw. von den Maßnahmen zur Eindämmung der Ausbreitung des Coronavirus stark betroffen sind, wie z. B. das Gastgewerbe, der Einzelhandel und weitere personenbezogene Dienstleistungen. Hinzu kann kommen, dass der Mindestlohn bzw. dessen Erhöhungen den Spielraum für Lohnanpassungen im unteren Lohnbereich einschränkt (Börschlein und Bossler, 2021). Insgesamt ist daher zu erwarten, dass vom Mindestlohn betroffene Betriebe häufiger mit kostenreduzierenden Personalmaßnahmen auf die Pandemie reagieren.

Laut bisherigen Studien hatte der gesetzliche Mindestlohn seit seiner Einführung nur geringe Auswirkungen auf die Beschäftigung in Deutschland (Pestel et al., 2020; Bossler et al., 2020). Allerdings waren durchaus verschiedene Anpassungsreaktionen bzw. Konsequenzen auf der betrieblichen Ebene zu beobachten. Zu den wichtigsten von Betrieben genannten Reaktionen gehörten die Erhöhung von Preisen und die Reduzierung von Arbeitszeiten (Bellmann et al., 2016; Statistisches Bundesamt, 2018; Mindestlohnkommission, 2020). Kausale Evaluationsstudien ergaben unter anderem, dass es mindestlohnbedingt zu einem Rückgang von Gewinnen und einem kurzfristigen Rückgang von Investitionen in Sachkapital kam (Bossler et al., 2020). Es zeigten sich bislang keine Auswirkungen des gesetzlichen Mindestlohns auf die betriebliche Produktivität (Bossler et al., 2020). Während sich insgesamt kein Effekt des Mindestlohns auf Betriebsschließungen zeigte (Bossler et al., 2018), deuten einige Befunde auf eine mindestlohnbedingte Zunahme von Betriebsschließungen bei Kleinstbetrieben mit bis zu zwei abhängig Beschäftigten hin (Dustmann et al., 2020).

* Der Artikel gibt ausschließlich die Meinung der Autoren und nicht notwendigerweise die der Mindestlohnkommission wieder.

1 2019 betrug der Mindestlohn 9,19 Euro und seit Januar 2020 9,35 Euro. Anfang 2021 stieg der gesetzliche Mindestlohn in Deutschland auf 9,50 Euro und seit Juli 2021 beträgt er 9,60 Euro.

**Christian Kagerl** ist wissenschaftlicher Mitarbeiter der Forschungsgruppe des Direktors am Institut für Arbeitsmarkt- und Berufsforschung (IAB) in Nürnberg.

Aktuell hat die Corona-Pandemie vielfältige Auswirkungen auf Beschäftigte und Betriebe in Deutschland. Unter anderem zeigte sich, dass Erwerbstätige mit geringeren Einkommen und mit niedrigerem Bildungsstand relativ stark von Arbeitszeitreduktion und Gehaltseinbußen betroffen waren (Kohlrausch et al., 2020; Schröder et al., 2020).[2] Dies erklärt sich teilweise dadurch, dass Dienstleistungsbereiche mit einem hohen Anteil von Niedriglohnbeschäftigten stark von den einschränkenden Maßnahmen gegen die Pandemie betroffen waren. Besonders der Handel (drei von zehn Betrieben) und das Gastgewerbe (neun von zehn Betrieben) waren zwischen Januar und April 2021 massiv vom Lockdown betroffen, im Gastgewerbe gab in diesem Zeitraum sogar ein Drittel aller Betriebe eine vollständige Einstellung der Geschäftsaktivität an (IAB, 2021). Hinzu kommt, dass Tätigkeiten mit niedriger Entlohnung häufig nicht für mobile Arbeit bzw. die Arbeit aus dem Homeoffice geeignet sind und bei entsprechenden Beschäftigten Kontaktbeschränkungen häufiger zu Kurzarbeit führen. Geringfügig Beschäftigte, die etwa die Hälfte der Mindestlohnbeziehenden ausmachen, haben allerdings keinen Anspruch auf Kurzarbeitergeld und waren von Einkommens- und Jobverlusten besonders stark betroffen (Grabka et al., 2020). Es stellt sich somit die Frage, ob Betriebe mit zum Mindestlohn beschäftigten Arbeitnehmer:innen spezifische Reaktionen auf die Corona-Pandemie zeigen. Dies kann darin begründet sein, dass Niedriglohnbetriebe von der Corona-Pandemie stärker betroffen sind oder grundsätzlich andere Eigenschaften und Dynamiken aufweisen als Betriebe mit höherem Lohnniveau. Außerdem wäre möglich, dass der Mindestlohn in der (Corona-)Krise eine spezifische Wirkung entfaltet. Die vorliegenden Befunde stellen die Situation der Mindestlohnbetriebe Anfang 2021 dar, ohne die Ursachen im Einzelnen identifizieren zu können.

## Datengrundlage

Die Betriebsbefragung „Betriebe in der Covid19-Krise" befragt seit August 2020 in ungefähr monatlichem Rhythmus 1.500 bis 2.000 Betriebe zu ihrem Umgang mit den zahlreichen Auswirkungen der Pandemie.[3] Die Stichprobe entstammt der Betriebsdatei der Bundesagentur für Arbeit, und die gewichteten Ergebnisse sind repräsentativ für die Gesamtheit der privatwirtschaftlichen Betriebe in Deutschland (Aminian et al., 2021). Die nachfolgenden Zahlen basieren auf der achten Welle der Befragung, an der zwischen dem 18. und 29. Januar 2021 1.997 Betriebe teilgenommen haben. Als vom Mindestlohn betroffen gelten im Rahmen der Befragung Betriebe, die Anfang

Abbildung 1
**Mindestlohnbetriebe und Betriebe mit negativen Corona-Auswirkungen nach Branchen**

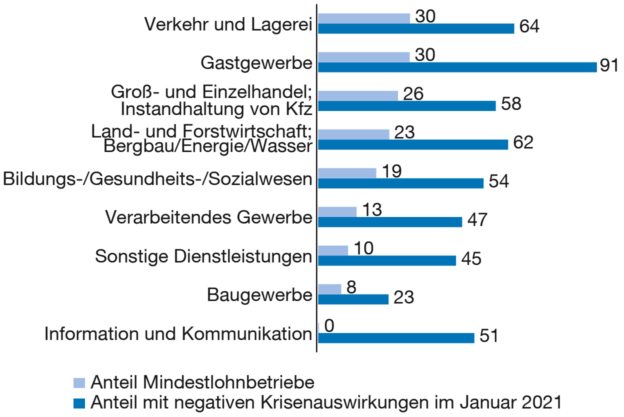

Anmerkung: Werte auf Basis von 1.997 befragten Betrieben.

Quelle: Betriebsbefragung „Betriebe in der Covid-19-Krise", Welle 8 (Jan 2021); eigene Berechnungen.

2021 mindestens einen Beschäftigten hatten, der zum Mindestlohn entlohnt wurde.[4]

## Betriebliche Betroffenheit vom Mindestlohn

Anhand der IAB-Befragung zeigt sich, dass zu Beginn des Jahres 2021 rund 17 % der Betriebe mindestens einen Beschäftigten hatten, der zum Mindestlohn entlohnt wurde. Der Anteil lag bei rund 20 % in Ostdeutschland und bei rund 16 % in Westdeutschland. Typischerweise ist die Mindestlohnbetroffenheit in Dienstleistungsbranchen besonders hoch (vgl. Abbildung 1). Dies betrifft insbesondere das Gastgewerbe, den Einzelhandel und den Bereich Logistik. Diese Branchen sind überwiegend durch direkten Kundenkontakt gekennzeichnet und tätigkeitsbedingt wenig für das Arbeiten von zu Hause geeignet. Dementsprechend sind die vom Mindestlohn stark betroffenen Branchen teilweise auch von der Corona-Pandemie besonders häufig negativ betroffen (Börschlein und Bossler, 2021). Die in Mindestlohnbetrieben beobachteten Entwicklungen 2020 und 2021 sind daher stark durch die Pandemie geprägt und gehen nicht oder nur zum Teil auf Auswirkungen des gesetzlichen Mindestlohns zurück. Mindestlohn bzw. Niedriglohnbetriebe können sich zudem auch in weiteren Merkmalen sowie ihrer Dynamik systematisch von Betrieben mit höherem Lohnniveau unterscheiden (Ohlert, 2021; Dütsch und Himmelreicher, 2020).

Insgesamt gaben 52 % der Betriebe an, negativ von der Corona-Pandemie betroffen zu sein. Nur 5 % nahmen positive Auswirkungen der Pandemie wahr. In weiteren 18 % hielten sich positive und negative Auswirkungen die Waa-

2 Dies gilt darüber hinaus auch für Selbstständige mit höheren Verdiensten (Grabka, 2021).

3 Die Erhebung wird durch das Institut für Arbeitsmarkt- und Berufsforschung (IAB) in Kooperation mit der Bundesanstalt für Arbeitsschutz und Arbeitsmedizin (BAuA) durchgeführt.

4 Die Frage lautete: „Gibt es in Ihrem Betrieb Beschäftigte, die zum gesetzlichen Mindestlohn von derzeit 9,50 Euro pro Stunde entlohnt werden?"

Abbildung 2
**Unterschiede in der Krisenbetroffenheit zwischen Mindestlohnbetrieben und anderen Betrieben**

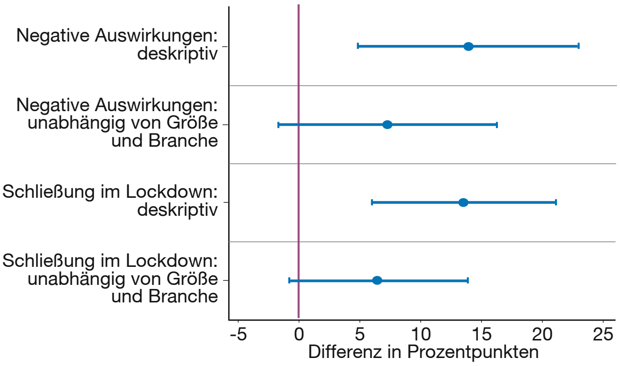

Anmerkung: durchschnittliche marginale Effekte und zugehörige 95 %-Konfidenzintervalle aus gewichteten logistischen Regressionen auf Basis von 1.997 befragten Betrieben.

Quelle: Betriebsbefragung „Betriebe in der Covid-19-Krise", Welle 8 (Jan 2021); eigene Berechnungen.

Abbildung 3
**Art der negativen Krisenauswirkungen auf Betriebe**

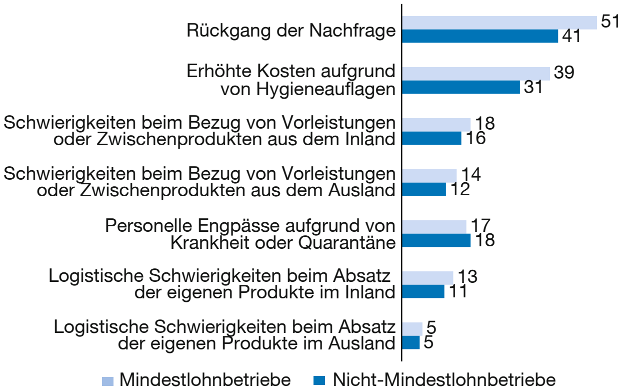

Anmerkung: Anteil der Betriebe mit mittleren oder schweren Auswirkungen der jeweiligen Art an allen Betrieben (Ausprägungen 3, 4 oder 5 auf einer Fünfer-Likert-Skala), Werte auf Basis von 1.997 befragten Betrieben.

Quelle: Betriebsbefragung „Betriebe in der Covid-19-Krise", Welle 8 (Jan 2021); eigene Berechnungen.

ge, während sich 25 % der Betriebe nicht betroffen sahen. Vom Mindestlohn betroffene Betriebe sind laut Selbsteinschätzung der Betriebe häufiger negativ von der Corona-Pandemie beeinflusst als Betriebe ohne Mindestlohnbeschäftigte. Die Mindestlohnbetriebe sind zu etwa 14 Prozentpunkten häufiger negativ betroffen. Kontrolliert man für die Zusammensetzung der Betriebe nach Branchen und Betriebsgrößen, beträgt dieser Unterschied noch rund 7 Prozentpunkte und ist nicht mehr statistisch signifikant. Die höhere Betroffenheit der Mindestlohnbetriebe ergibt sich also zu einem beträchtlichen Teil daraus, dass diese in krisenbetroffenen Branchen wirtschaftlich aktiv sind. Dieser Sachverhalt ist sehr ähnlich, wenn anstatt der allgemeinen Einschätzung zu negativen Auswirkungen betrachtet wird, ob Betriebe wegen der Verfügungen des Lockdowns im Januar 2021 zumindest teilweise schließen mussten (vgl. Abbildung 2). Für eine höhere Betroffenheit der Mindestlohnbetriebe durch die Corona-Pandemie spricht zudem, dass diese Betriebe häufiger staatliche Förderungen zur Überbrückung in Anspruch nahmen. Im Januar 2021 hatte knapp ein Viertel (23 %) der Mindestlohnbetriebe die Überbrückungshilfe der Bundesregierung für das vierte Quartal 2020 bereits beantragt oder plante, dies zu tun. Bei den Nicht-Mindestlohnbetrieben waren es lediglich 13 %. Anhand der IAB-Erhebung bestätigt sich auch, dass die Möglichkeit für Homeoffice in Mindestlohnbetrieben (32 %) weniger verbreitet ist als bei anderen Betrieben (49 %).

Anhand der Befragung lässt sich auch ermitteln, welche spezifischen Schwierigkeiten die Corona-Pandemie bei Betrieben im Januar 2021 hervorrief. Dabei nannten die Betriebe am häufigsten einen Rückgang der Nachfrage sowie erhöhte Kosten aufgrund von Hygieneauflagen (vgl. Abbildung 3). Beides war bei Mindestlohnbetrieben deutlich häufiger der Fall als bei Nicht-Mindestlohnbetrieben. Z. B. waren 39 % der Mindestlohnbetriebe von Kosten durch die allgegenwärtigen Hygieneauflagen betroffen, wohingegen dies nur auf 31 % der restlichen Betriebe zutraf. Trotz dieser beobachtbaren Tendenzen sind die Differenzen nicht statistisch signifikant (mit und ohne Berücksichtigung von Kontrollvariablen). Beim Absatz der eigenen Produkte und beim Bezug von Vorleistungen zeigen sich deskriptiv keine nennenswerten Unterschiede. Es wäre denkbar, dass Mindestlohnbetriebe nicht nur häufiger, sondern auch stärker von der Pandemie betroffen sind als andere Betriebe. Die IAB-Erhebung zeigt allerdings bei Betrieben, die grundsätzlich negativ von der Krise betroffen sind, keine Unterschiede in der Stärke der Betroffenheit.

## Betriebliche Reaktionen auf die Corona-Pandemie

Um auf die Krise zu reagieren, haben Unternehmen zahlreiche Möglichkeiten. Zur Vermeidung von Entlassungen hat sich während der Pandemie das Instrument der Kurzarbeit als äußerst relevant erwiesen. Doch auch über die Kurzarbeit hinaus stehen weitere Maßnahmen zur Verfügung, eine als temporär empfundene Krise ohne Entlassungen zu überwinden (Bellmann et al., 2020). Änderungen in der Lohn- und Gehaltspolitik oder Maßnahmen wie der Abbau von Überstunden kommen hier infrage (Kagerl und Bruttel, 2021).

### Beschäftigungsdynamik und Kurzarbeit

Nur rund 5 % der Betriebe gaben an, Anfang 2021 (in den letzten drei Wochen vor der Befragung) Beschäftigte entlassen zu haben. Von diesen Betrieben gaben rund 41 %

Abbildung 4
**Arbeitszeitmaßnahmen in der Corona-Krise**

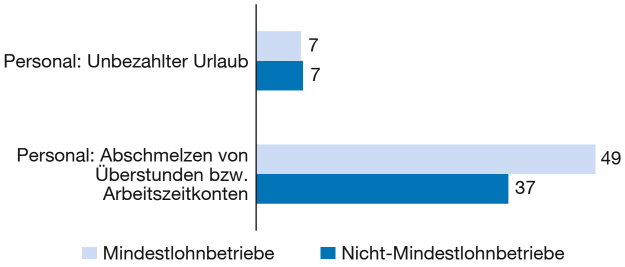

Anmerkung: Werte auf Basis von 1.997 befragten Betrieben.

Quelle: Betriebsbefragung „Betriebe in der Covid-19-Krise", Welle 8 (Jan 2021; eigene Berechnungen.

an, Entlassungen aufgrund der Corona-Pandemie vorgenommen zu haben. Diese Angaben unterscheiden sich nicht signifikant zwischen Mindestlohnbetrieben und der sonstigen Wirtschaft. Allerdings gaben 32 % der Mindestlohnbetriebe an, seit Beginn der Krise auf Neueinstellungen verzichtet zu haben, während dies nur 29 % der restlichen Betriebe taten, obschon diese Differenz nicht statistisch signifikant ist. Diese Tendenz deckt sich mit Ergebnissen zu einem zwischenzeitlichen deutlichen Rückgang offener Stellen 2020, der ebenfalls bei Mindestlohnbetrieben stärker ausfiel (Börschlein und Bossler, 2021).

Die Nutzung von Kurzarbeit kann die Personalkosten für krisenbetroffene Betriebe reduzieren und somit negative Beschäftigungseffekte verhindern. Bei etwa 23 % der Betriebe war im Januar 2021 mindestens ein Teil der Belegschaft in Kurzarbeit. Der Anteil war mit rund 29 % in vom Mindestlohn betroffenen Betrieben um 7 Prozentpunkte höher als in den übrigen Betrieben. Der Unterschied ist allerdings nicht statistisch signifikant. Eine signifikant höhere Intensität der Kurzarbeit (in % der Belegschaft) lässt sich auf Basis der Befragung ebenfalls nicht feststellen, in kurzarbeitenden Mindestlohnbetrieben waren im Januar durchschnittlich 55 % der Belegschaft in Kurzarbeit, in anderen kurzarbeitenden Betrieben waren es 59 %. Für das 3. Quartal 2020 zeigte sich anhand der IAB-Stellenerhebung eine signifikant höhere Nutzung von Kurzarbeit in Mindestlohnbetrieben (Börschlein und Bossler, 2021).

### Arbeitszeitmaßnahmen

Neben der Kurzarbeit kommen weitere arbeitszeitreduzierende Maßnahmen in Betracht, um in der Krise Personalkosten zu sparen. Unbezahlter Urlaub wurde insgesamt nur in wenigen Betrieben aufgrund der Corona-Krise genommen. Das Abschmelzen von Überstunden bzw. Arbeitszeitkonten aufgrund der Krise war allerdings in Mindestlohnbetrieben signifikant häufiger verbreitet als in Nicht-Mindestlohnbetrieben (vgl. Abbildung 4).

Abbildung 5
**Lohn- und Gehaltspolitik in der Corona-Krise**

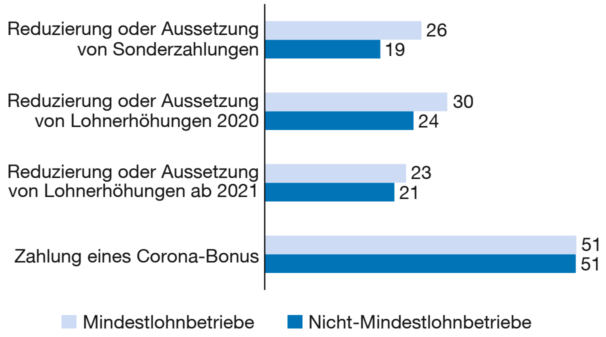

Anmerkung: Werte auf Basis von 1.997 befragten Betrieben.

Quelle: Betriebsbefragung „Betriebe in der Covid-19-Krise", Welle 8 (Jan 2021); eigene Berechnungen.

### Lohn- und Gehaltspolitik

Vom Mindestlohn betroffene Betriebe könnten spezifische Maßnahmen treffen, um auf die Corona-Pandemie zu reagieren. Der Mindestlohn beschränkt für diese Betriebe grundsätzlich die Lohnanpassungsmöglichkeiten nach unten hin. Allerdings ist davon auszugehen, dass nominale Lohnreduzierungen selbst in der Krise nur selten vorkommen. Laut einer Befragung würden nur etwa 2 % aller Betriebe gerne Stundenlöhne unter das Niveau des Mindestlohns senken, rund 4 % der Betriebe gaben an, eine Neueinstellung unterhalb des Mindestlohnniveaus tätigen zu wollen (Börschlein und Bossler, 2021). Dies könnte sich in Bezug auf Erhöhungen des Mindestlohns anders darstellen. Denn Lohnerhöhungen würden Unternehmen in Krisenzeiten unter Umständen durchaus zurückstellen.

Die Ergebnisse aus der Erhebung zeigen, dass Mindestlohnbetriebe 2020 tendenziell häufiger Lohnerhöhungen reduziert oder ausgesetzt haben als andere Betriebe (vgl. Abbildung 5). Für 2021 war dies durch Mindestlohnbetriebe ebenfalls häufiger vorgesehen. Diese Unterschiede sind allerdings nicht statistisch signifikant. Reduzierungen oder die Aussetzung von Sonderzahlungen hingegen wurden durch Mindestlohnbetriebe signifikant häufiger vorgenommen als durch andere Betriebe. Die Zahlung eines Corona-Bonus wurde dagegen bei etwa der Hälfte sowohl der Mindestlohnbetriebe als auch anderer Betriebe durchgeführt.

## Fazit

Mindestlohnbetriebe sind besonders oft Betriebe kleiner und mittlerer Größe in Gastronomie, Einzelhandel, Logistik oder weiteren Dienstleistungsbranchen. Die Arbeit in diesen Branchen ist überwiegend durch direkten Kundenkontakt gekennzeichnet und daher häufiger von Hygieneauflagen betroffen und tätigkeitsbedingt weniger für das Arbeiten von zu

Hause geeignet. Es ist daher davon auszugehen, dass Betriebe mit zum Mindestlohn Beschäftigten die Corona-Pandemie besonders deutlich zu spüren bekommen. Der Mindestlohn beschränkt grundsätzlich die Lohnanpassungsmöglichkeiten nach unten. Im Zeitraum der Pandemie stieg der Mindestlohn allerdings nur moderat, von 9,19 Euro 2019 auf 9,35 Euro 2020 und auf 9,50 Euro zu Beginn des Jahres 2021. Die achte Welle der Erhebung „Betriebe in der Covid-19-Krise“ des IAB fand im Januar 2021 statt und erhob neben der Situation in der Pandemie auch, ob die Betriebe Mindestlohnbeschäftigte hatten. Aufgrund des Fragezeitpunkts beziehen sich die Angaben überwiegend auf den (bisherigen) Höhepunkt der Corona-Pandemie im Winter 2020/2021.

Die Ergebnisse zeigen, dass Mindestlohnbetriebe zu Beginn 2021 häufiger von der Corona-Pandemie betroffen waren als die restliche Wirtschaft. Dies geht insbesondere auf die Branchenzusammensetzung der Mindestlohnbetriebe zurück. Die Befunde deuten des Weiteren darauf hin, dass Mindestlohnbetriebe etwas häufiger Kurzarbeit in Anspruch nehmen als andere Betriebe. Der Arbeitsumfang wird in Mindestlohnbetrieben auch durch den Abbau von Überstunden häufiger reduziert als in anderen Betrieben. Zudem werden Gehaltserhöhungen und Sonderzahlungen häufiger reduziert oder ausgesetzt. Entlassungen kamen Anfang 2021 insgesamt, wie auch bei den Mindestlohnbetrieben nur bei einem kleinen Teil von Betrieben vor. Offensichtlich konnten die Kurzarbeit sowie die weiteren Personal- und Lohnmaßnahmen einen Beschäftigungsabbau effektiv verhindern.

Die Befunde über die Situation der Mindestlohnbetriebe spiegeln wider, dass sich die Auswirkungen der Pandemie in manchen Wirtschaftsbereichen und Beschäftigungssegmenten ballen. Demnach sind sowohl Erwerbstätige mit geringeren Einkommen und mit niedrigerem Bildungsstand (Kohlrausch et al., 2020; Schröder et al., 2020), als auch Betriebe, die geringe Löhne zahlen, vergleichsweise stark negativ von der Krise betroffen. Entsprechend nutzten Mindestlohnbetriebe auch häufiger als andere Betriebe die Überbrückungshilfe der Bundesregierung. Die Situation von Mindestlohnbetrieben in der Corona-Pandemie hängt maßgeblich mit deren Strukturmerkmalen zusammen und geht nach bisherigen Erkenntnissen nicht ursächlich auf den Mindestlohn zurück.

**Title:** ***Minimum Wage Companies in the Second Wave of the Corona Crisis***

**JEL Classification:** *J23, J38, M54*
